# Transpulmonary thermodilution detects rapid and reversible increases in lung water induced by positive end-expiratory pressure in acute respiratory distress syndrome

**DOI:** 10.1186/s13613-020-0644-2

**Published:** 2020-03-02

**Authors:** Francesco Gavelli, Jean-Louis Teboul, Danila Azzolina, Alexandra Beurton, Temistocle Taccheri, Imane Adda, Christopher Lai, Gian Carlo Avanzi, Xavier Monnet

**Affiliations:** 1 Service de médecine intensive - réanimation, Hôpitaux universitaires Paris-Saclay, Hôpital de Bicêtre, APHP, 78, rue du Général Leclerc, 94270 Le Kremlin-Bicêtre, France; 2 Inserm UMR S_999, Univ Paris-Saclay, 78, rue du Général Leclerc, 94270 Le Kremlin-Bicêtre, France; 3 Dipartimento di Medicina Traslazionale, Università del Piemonte Orientale, Via Solaroli 17, 28100 Novara, Italy

**Keywords:** Pulmonary oedema, Pulmonary lymphatic drainage, Central venous pressure, Lung recruitment, Mechanical ventilation

## Abstract

**Purpose:**

It has been suggested that, by recruiting lung regions and enlarging the distribution volume of the cold indicator, increasing the positive end-expiratory pressure (PEEP) may lead to an artefactual overestimation of extravascular lung water (EVLW) by transpulmonary thermodilution (TPTD).

**Methods:**

In 60 ARDS patients, we measured EVLW (PiCCO2 device) at a PEEP level set to reach a plateau pressure of 30 cmH_2_O (HighPEEP_start_) and 15 and 45 min after decreasing PEEP to 5 cmH_2_O (LowPEEP_15′_ and LowPEEP_45′_, respectively). Then, we increased PEEP back to the baseline level (HighPEEP_end_). Between HighPEEP_start_ and LowPEEP_15′_, we estimated the degree of lung derecruitment either by measuring changes in the compliance of the respiratory system (Crs) in the whole population, or by measuring the lung derecruited volume in 30 patients. We defined patients with a large derecruitment from the other ones as patients in whom the Crs changes and the measured derecruited volume were larger than the median of these variables observed in the whole population.

**Results:**

Reducing PEEP from HighPEEP_start_ (14 ± 2 cmH_2_O) to LowPEEP_15′_ significantly decreased EVLW from 20 ± 4 to 18 ± 4 mL/kg, central venous pressure (CVP) from 15 ± 4 to 12 ± 4 mmHg, the arterial oxygen tension over inspired oxygen fraction (PaO_2_/FiO_2_) ratio from 184 ± 76 to 150 ± 69 mmHg and lung volume by 144 [68–420] mL. The EVLW decrease was similar in “large derecruiters” and the other patients. When PEEP was re-increased to HighPEEP_end_, CVP, PaO_2_/FiO_2_ and EVLW significantly re-increased. At linear mixed effect model, EVLW changes were significantly determined only by changes in PEEP and CVP (*p* < 0.001 and *p* = 0.03, respectively, *n* = 60). When the same analysis was performed by estimating recruitment according to lung volume changes (*n* = 30), CVP remained significantly associated to the changes in EVLW (*p* < 0.001).

**Conclusions:**

In ARDS patients, changing the PEEP level induced parallel, small and reversible changes in EVLW. These changes were not due to an artefact of the TPTD technique and were likely due to the PEEP-induced changes in CVP, which is the backward pressure of the lung lymphatic drainage.

*Trial registration* ID RCB: 2015-A01654-45. Registered 23 October 2015

## Background

Extravascular lung water (EVLW) is the amount of fluid present in the lungs, outside the pulmonary blood vessels [1]. In acute respiratory distress syndrome (ARDS), lung injury leads to increases in the pulmonary capillary permeability and in EVLW, which reflect the severity of the disease [2].

Many studies have investigated the changes in EVLW induced by a positive end-expiratory pressure (PEEP), which is the cornerstone of ARDS treatment (Additional file 1: Table S1). However, they have provided very discordant results, some showing that EVLW augmented when increasing levels of PEEP were applied [3–8], some that it decreased [9–17] and some others that it did not change [18–28].

The large majority of these studies were conducted in animals [3–5, 8–27], with various models of ARDS and methods of EVLW estimation. Today, the routine measurement of EVLW at the bedside is allowed by transpulmonary thermodilution (TPTD). One animal [8] and three human studies [6, 7, 28] have investigated the effects of PEEP changes on TPTD-estimated EVLW, three suggesting that EVLW increases with PEEP [6–8] and another one that it remains unchanged [28]. However, these studies did not investigate the potential artefact that may induce an increase in EVLW along with the PEEP level.

Indeed, the PEEP-induced lung recruitment may relieve the hypoxic vasoconstriction of the recruited regions, which eventually become accessible to the cold indicator while they were not at a lower PEEP level. This may lead to an artefactual overestimation of the PEEP-induced EVLW augmentation.

Thus, the goal of our study, conducted in ARDS patients, was to investigate whether the estimation of EVLW by TPTD is artefactually influenced by the lung derecruitment potentially secondary to the decrease in the PEEP level.

## Methods

### Patients

This prospective, one-centre study was approved by the Institutional Review Board of our institution (Comité pour la Protection des Personnes, Ile-de-France VII, IDCRB 2015-A01654-45). At the time of inclusion, patients’ relatives were informed of the study protocol and possibility was given to them to refuse participation. As soon as clinical condition improved and patients were able to give consent, the same information was delivered to them, with possibility for them to deny the participation. All patients and/or relatives accepted to participate.

Inclusion criteria were age ≥ 18 years, presence of ARDS according to the Berlin definition [29] and monitoring with a TPTD device (PiCCO2 device, Pulsion Medical Systems, Feldkirchen, Germany). Exclusion criteria were contraindications to PEEP increase (pneumothorax, uncontrolled shock state) and extracorporeal membrane oxygenation, which impedes the measurement of EVLW by TPTD. Patients could be under continuous venovenous haemofiltration since it does not affect the TPTD estimation of EVLW [30, 31].

### TPTD measurements

TPTD measurements were performed by injecting 15-mL boluses of saline (< 8 °C) through a jugular vein catheter. In order to allow the detection of small changes in EVLW, the average of the results obtained by five successive thermodilution measurements was used. With this number of replicates, the least significant change of EVLW is 8% [32].

With TPTD, we also measured the pulmonary vascular permeability index (PVPI) [1, 33] (also averaged from five successive thermodilution measurements) and cardiac index (CI).

### Haemodynamic measurements

In addition to arterial pressure and CI, we measured the central venous pressure (CVP) at the base of the C wave, at end-expiration. The value of three successive respiratory cycles was averaged. The pressure transducer was attached to the arm, at a height corresponding to the level of the right atrium.

### Estimation of alveolar derecruitment induced by PEEP decreases

In a subgroup of 30 patients ventilated with an Infinity V500 ventilator (Dräger, Lübeck, Germany), we directly estimated the volume of derecruited lung during the PEEP decrease. For this purpose, after transiently reducing the respiratory rate to 10 breaths/min to reduce the risk of air trapping, a prolonged expiration was performed while abruptly reducing PEEP from its baseline value to 5 cmH_2_O for one breath. The difference in end-expiratory Vt between the breath while PEEP was decreased and the one before was defined as the total change in lung volume [34]. At the same time, we estimated the minimal predicted change in lung volume determined by the PEEP change, as previously described [35]. Briefly, the respiratory system compliance at low PEEP was multiplied by the pressure difference between the two PEEP levels. Then, this value was subtracted from the total change in lung volume and the result was considered as an estimation of derecruited lung volume induced by PEEP reduction [34, 35]. In addition, in the whole population, we estimated the degree of derecruitment during the PEEP decrease by observing the simultaneous changes in compliance of the respiratory system (Crs) [36]. For this purpose, Crs was calculated as the ratio of tidal volume (Vt) over the driving pressure (plateau pressure—PEEP).

We defined patients with a large derecruitment from the other ones as patients in whom Crs changes and the measured derecruited volume were larger than the median of their value observed in the whole population.

### Study design

At baseline, patients were ventilated in the assist-control mode with a Vt at 6 mL/kg (predicted body weight). PEEP was set to reach a plateau pressure of 28–30 cmH_2_O (High-PEEP) [37]. Sedation was provided by propofol and remifentanil.

At this time (High-PEEP_start_), a first set of measurements was performed including heart rate, arterial pressure, CVP, EVLW and blood gas analysis. PEEP was then decreased, while the derecruited volume was estimated in the 30 patients in whom it was possible. After 15 min (Low-PEEP_15′_) and 45 min (Low-PEEP_45′_), we measured the same variables as at baseline. A time interval of 45 min appeared to us as reasonably long enough for allowing potential fluid transfer through the pulmonary capillary barrier. Thereafter, PEEP was increased back to its baseline level. After 15 min, the variables measured at baseline were measured again (High-PEEP_end_).

Sedative drugs, Vt, respiratory rate, and the fraction of inspired oxygen (FiO_2_) remained unchanged during the study. Volume expansion, fluid removal, recruitment manoeuvres, administration of inhaled nitric oxide or nebulization were not performed during this time.

### Statistical analysis

Considering an *α* risk of 5% and a *β* risk of 20%, to evidence a PEEP-induced change in EVLW by 2 ± 4 mL/kg, we estimated that 54 patients should be included into the study, a number that was rounded to 60. The PEEP-induced change in EVLW was estimated by considering that the least significant change of the measurement is 8% if five values of TPTD are averaged [32] and by expecting a baseline EVLW of 20 ± 6 mL/kg [38].

Data are expressed as mean ± standard deviation for normally distributed variables or median [interquartile range] for skewed data. A Shapiro–Wilk test was considered to determine if a variable was well-modelled by a normal distribution. The analysis of patients with a large derecruitment compared to the other ones was planned a priori.

A linear mixed factor ANOVA for repeated measurements was used to evaluate both within-subject effect (PEEP/time effect) and between-subject effects (recruiting effect). Both High-PEEP_start_ and Low-PEEP_45′_ have been considered as reference categories for comparisons. Multiple comparisons of means have been performed using Tukey contrasts.

The covariate effect on EVLW outcome was then estimated using a linear mixed model for repeated measurements (random intercept model) adjusting the estimates for PEEP, position (prone/supine) and recruiting effect according either to the Crs changes and the recruited lung volume. Sample size calculation and statistical analysis were performed with MedCalc 18.2.1 software (Mariakerke, Belgium) and R 3.5.2 statistical software with lme4 package.

## Results

### Patients

Sixty consecutive patients were included. On average, ARDS developed for 3 [1–5] days at the time of inclusion. Septic shock was present in 54 (90%) patients (Table 1). Pneumonia was the cause of ARDS in all patients. The number of chest X-ray quadrants involved was two in 21 (35%) cases, three in 35 (58%) cases and four in 4 (7%) patients. At baseline, blood lactate was 2.5 [1.6–3.4] mmol/L, creatinine 98 [66–106] μmol/L and 15 (25%) patients had renal replacement therapy in place (conventional haemodialysis in three, continuous venovenous hemofiltration in 12 patients, without weight loss). Eleven (18%) patients were in prone position at the time of inclusion, whereas 28 (47%) other ones had required prone positioning before the inclusion (Table 1). Seventeen (28%) patients were paralysed at the time of inclusion and the Richmond Agitation-Sedation Scale was − 4 [− 5 to − 3].

**Table 1:** Patient characteristics

Patient characteristics (*n* = 60)	
Age (years)	69 ± 10
Male gender (*n*, %)	34 (57%)
Body mass index (kg/m^2^)	25 ± 4
Simplified Acute Physiologic Score II on inclusion	51 ± 18
ARDS severity (*n*, %)	
Mild	21 (35%)
Moderate	31 (52%)
Severe	8 (13%)
Aetiology of ARDS (*n*, %)	
Community acquired pneumonia	46 (77%)
Aspiration pneumonia with neurologic disorders	5 (8%)
Ventilator associated pneumonia	9 (15%)
ICU length of stay (days)	12 [11–38]
Total duration of mechanical ventilation (days)	11 [10–37]
Mortality at day-28 (*n*, %)	26 (43%)
Norepinephrine	
Number of patients (%)	54 (90%)
Dose of norepinephrine (µg/kg/min)	0.53 [0.27–1.00]
Left ventricular ejection fraction (%)	44 ± 11
*E*/*e*′ ratio	9 ± 2
Ventilator settings	
Tidal volume (mL/kg of PBW)	5.5 [5.0–6.0]
Respiratory rate (breaths/min)	27 ± 5
Fraction of inspired oxygen	0.64 ± 0.20
Patients requiring prone position (*n*, %)	39 (65%)

ARDS: acute respiratory distress syndrome; *E*/*e*′: ratio of the amplitude of the *E* wave over the amplitude of the *e*′ wave of the mitral flow with echocardiography; ICU: intensive care unit; PBW: predicted body weight

### Effects of PEEP changes on haemodynamic variables

The decrease in PEEP from High-PEEP_start_ decreased CVP by 21 ± 13% (*p* < 0.01) (Table 2). When PEEP was increased from Low-PEEP_45′_ to High-PEEP_end_, opposite and symmetrical changes were observed (Table 2; see Additional file 2: Table S2 for post hoc comparisons).

**Table 2:** Haemodynamic and respiratory variables

Variables	High-PEEP_start_	Low-PEEP_15′_	Low-PEEP_45′_	High-PEEP_end_
Heart rate (min^−1^)	89 ± 18	90 ± 19	91 ± 19	91 ± 19
Systolic arterial pressure (mmHg)	130 ± 25	132 ± 19	129 ± 19	124 ± 17
Diastolic arterial pressure (mmHg)	65 ± 20	63 ± 10	62 ± 11	62 ± 10
Mean arterial pressure (mmHg)	86 ± 16	86 ± 13	85 ± 14	83 ± 11
Central venous pressure (mmHg)	15 ± 4	12 ± 4*	12 ± 4*	15 ± 4**
Cardiac index (L/min/m^2^)	2.77 ± 0.79	3.08 ± 0.85*	3.12 ± 0.90*	2.81 ± 0.89**
Cardiac function index (min^−1^)	4.0 ± 1.2	4.3 ± 1.3*	4.3 ± 1.3*	4.1 ± 1.3**
Global end-diastolic volume indexed (mL/m^2^)	750 ± 116	787 ± 168*	791 ± 150*	748 ± 127**
Extravascular lung water (mL/kg)	20 ± 4	18 ± 4*	18 ± 4*	20 ± 5**
Pulmonary vascular permeability index	3.6 ± 1.0	3.5 ± 1.0*	3.5 ± 1.0*	3.6 ± 0.9
Pulse pressure variation (%)	7 [4–14]	6 [4–12]	8 [4–12]	7 [5–11]
Stroke volume variation (%)	8 [5–13]	7 [4–13]	7 [5–13]	8 [6–13]
PEEP (cmH_2_O)	14 ± 2	5 ± 0*	5 ± 0*	14 ± 2**
Pplateau (cmH_2_O)	28 ± 2	20 ± 3*	20 ± 3*	28 ± 2**
Respiratory system compliance (mL/cmH_2_O)	27 [22–32]	26 [24–28]	25 [23–28]	27 [23–31]
SpO_2_ (%)	98 ± 2	96 ± 3*	95 ± 4*	98 ± 2**
SaO_2_ (%)	97 ± 3	94 ± 5*	93 ± 6+*	96 ± 4**
PaO_2_/FiO_2_ ratio	184 ± 76	150 ± 69*	147 ± 68*	178 ± 76**

*FiO*_*2*_ inspired oxygen fraction, *PEEP* positive end-expiratory pressure, *Pplateau* plateau pressure, *PaO*_*2*_ arterial oxygen partial pressure, *SaO*_*2*_ arterial oxygen saturation, *SpO*_*2*_ pulse oxygen saturation

*p < 0.05 vs. High-PEEP_start_, **p < 0.05 vs. Low-PEEP_45′_. See Additional file 2: Table S2 for post hoc comparisons

### Effects of PEEP changes on respiratory variables

From High-PEEP_start_ to Low-PEEP_15′_, PEEP decreased by 9 ± 2 cmH_2_O and this was accompanied by a decrease in the plateau pressure by 8 ± 3 cmH_2_O. When decreasing PEEP from High-PEEP_start_, the change in Crs was 0.0 [− 3.8 to 3.8] mL/cmH_2_O (*n* = 60) (Table 2). Since the median value of Crs changes was 0.0 mL/cmH_2_O, we defined derecruiters as patients in whom decreasing PEEP induced a decrease in Crs. When decreasing PEEP from High-PEEP_start_, the estimated derecruited lung volume was 144 [68–420] mL in the patients in whom it was measured (*n* = 30).

No differences were found in terms of EVLW changes between derecruiters and the other patients, defined according to either the Crs change (*n* = 60) or the derecruited volume (*n* = 30). All the significant changes in respiratory variables reversed with a similar amplitude when PEEP was increased from Low-PEEP_45′_ to High-PEEP_end_ (Table 2).

### Effects of PEEP changes on EVLW

**Fig. 1:**
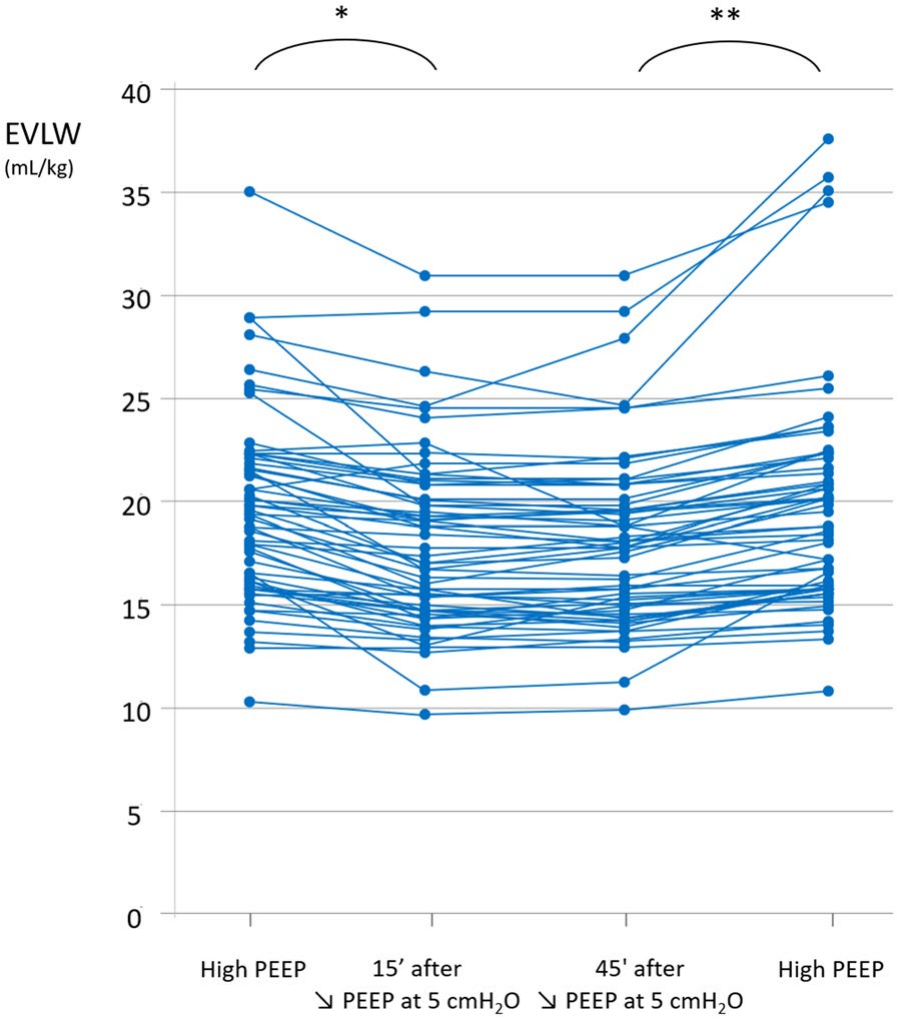
Individual values of extravascular lung water (EVLW) at different study times. *PEEP* positive end-expiratory pressure. *p < 0.05 vs. High-PEEP, **p < 0.05 vs. 45′ after decreasing PEEP at 5 cmH_2_O

Decreasing PEEP from High-PEEP_start_ induced a significant decrease in EVLW by 8 ± 7% (*p* < 0.01) (Table 2, Fig. 1). This decrease in EVLW was observed in all the patients but two (Fig. 1). It persisted at Low-PEEP_15′_ and Low-PEEP_45′_. When PEEP was increased from Low-PEEP_45′_ to High-PEEP_end_, opposite and symmetrical changes in EVLW were observed (Table 2).

When we evaluated the covariate effect on EVLW at the linear mixed model for repeated measures, adjusted for PEEP, prone/supine position and recruitment according to Crs changes, only the changes in PEEP and CVP were significantly associated to the changes in EVLW (*p* < 0.001 and *p* = 0.03, respectively) (Table 3). When we performed the same analysis by estimating recruitment according to lung volume changes (*n* = 30), CVP but not the recruited lung volume remained significantly associated to the changes in EVLW (*p* < 0.001).

**Table 3:** Linear mixed model estimation on extravascular lung water changes adjusted by presence or absence of prone position and by changes in positive end-expiratory pressure, central venous pressure, and compliance of the respiratory system

	Value	Standard error	DF	*t* value	*p* value
Intercept	15.58	1.15	178	13.53	
CVP	0.15	0.07	178	2.16	0.03
PEEP	0.13	0.03	178	4.79	< 0.01
Prone position	− 0.78	1.56	57	− 0.5	0.62
Crs	0.61	1.21	57	0.5	0.62

*CVP* central venous pressure, *DF* degrees of freedom, *PEEP* positive end-expiratory pressure, *Crs* respiratory system compliance

## Discussion

This study shows that decreasing PEEP in ARDS patients induces a small, reversible and rapid decrease in EVLW measured by TPTD. The recruited lung volume was not independently associated with this change in EVLW, while it was the case for the change in CVP.

### Were the PEEP-induced changes in EVLW due to artefacts of the TPTD technique?

At the bedside, the only technique that allows the measurement of EVLW is TPTD. Although it can detect interstitial oedema, lung ultrasound does not allow the quantification of the EVLW total volume, and CT scan cannot be used routinely. The estimation of EVLW by TPTD in humans has been demonstrated to correlate with the one provided by gravimetry [39], which is the reference technique. Even small and rapid changes in EVLW can be measured [40]. The value of EVLW has been regularly demonstrated to be correlated with mortality in critically ill patients [41, 42], especially in septic shock [43, 44] and ARDS [38, 45].

Nevertheless, the ability of TPTD to assess the changes in EVLW induced by PEEP has been only scarcely investigated, despite its important role in ARDS management [46]. Moreover, the few available studies did not specifically investigate the artefact that may affect the TPTD estimation of PEEP-induced changes in EVLW [6–8, 28]. As a matter of fact, by relieving the hypoxic vasoconstriction in recruited areas, PEEP may allow the cold indicator to reach these regions, increasing the volume of EVLW that is accessible to measurement. In our study, the changes in EVLW were the same among patients with high or low derecruitment, when derecruitment was assessed by the PEEP-induced change in lung volume, the method that is today the best one for estimating recruitment/derecruitment at the bedside [36]. It was also the same in the whole population, when we defined derecruitment as a decrease in Crs. Moreover, neither the estimated derecruited lung volume nor the Crs changes were independently associated with EVLW changes at linear mixed model analysis.

Another argument against the explanation of EVLW changes by artefacts due to lung recruitment is that the changes in EVLW were observed rapidly both after reducing and increasing the PEEP level. Incrementing and decrementing PEEP have different impact on the time required to reach equilibrium in the respiratory system [47]. The fact that specular changes were observed after opposite PEEP changes strongly suggests that a haemodynamic mechanism may be a more plausible explanation for the observed results.

### Mechanisms of the PEEP-induced changes in EVLW

Since the PEEP-induced changes in EVLW we observed were not due to artefacts in the TPTD estimation, one should consider that EVLW was really decreased when the PEEP level was reduced, and that this small and rapid change was reversible. Although of small amplitude, the EVLW changes were actually significant. Moreover, Fig. 1 shows well how EVLW changes were very consistent among patients. Also, we took the precaution to measure EVLW by averaging not three but five TPTD measurements, which enabled us to reliably detect small changes in EVLW [32].

Our results are in accordance with the previous studies which, amongst very discrepant ones, suggested that PEEP induces small increases in EVLW [3–8]. In theory, three mechanisms might explain why EVLW varies in the same direction as PEEP (Additional file 3: Figure S1). First, decreasing PEEP decreases CVP, which is the backward pressure of the drainage through the thoracic duct. This may happen by direct transmission of the intrathoracic pressure to the right atrial pressure, or as the result of the decrease in the right ventricular afterload. Although the changes in EVLW were of lower amplitude than those of CVP, the results of the linear mixed effect model make this pathophysiological hypothesis acceptable. Of note, even though it may increase CVP in ARDS patients [48, 49], prone position in our population was not an element influencing the relationship between CVP and EVLW.

The second mechanism which may explain why the PEEP decrease diminished EVLW is a decrease in the formation of lung water (Additional file 3: Figure S1). Indeed, the intrathoracic pressure is transmitted to the left atrium, such that when PEEP is decreased, the intramural pulmonary capillary pressure is decreased as well. It is well known that, on the opposite, augmenting PEEP increases the intramural pulmonary artery occlusion pressure [50]. We could not assess this mechanism, since we estimated neither the pulmonary capillary pressure nor the pulmonary artery occlusion pressure in our study.

The normal pulmonary lymphatic flow is estimated to be 8–9 mL/h in humans [51]. Nevertheless, it has been reported that the pulmonary lymphatic flow could increase to tenfold, or even more, during ARDS [52]. Moreover, the estimation of pulmonary lymphatic flow in humans comes from animal studies, and it is much of an assumption that lymph flow is the same per kilogram of bodyweight in humans as in dogs [51]. Then, this is compatible with the amount of changes in EVLW we observed. The PEEP decrease led to a reduction of EVLW by 1.6 ± 1.6 mL/kg, which was equivalent to roughly 100 mL of lung water accumulated in 15 min, and vice versa when PEEP was re-increased. Nevertheless, since we did not directly measure the lymphatic flow and since the link between EVLW and CVP observed in our results was imperfect, we cannot exclude the contribution of other mechanisms.

In particular, it might be possible that part of the changes in EVLW we observed were related to changes in lung permeability, although this seems to be unlikely in such a short time. The decrease in PEEP was associated with a significant but slight decrease in PVPI, which reflects alveolo-capillary permeability. Nevertheless, this change was very small, and was not significantly reversed when PEEP was re-increased.

### Practical implications

First, our findings show that TPTD is not flawed by the level of PEEP, as it has been previously suspected [53]. Second, our observation that increasing PEEP increases EVLW does not challenge the benefit of PEEP in ARDS. The increase in EVLW we report was small and might be easily counterbalanced by the potential benefits of PEEP such as increase in end-expiratory lung volume induced by recruitment, decrease in pulmonary shunt in recruiters and redistribution of alveolar fluid to extra-alveolar spaces [36]. Nevertheless, when using TPTD at the bedside [54], clinicians should be aware that changing PEEP might slightly change EVLW and that it is not due to a worsening of the disease or to the deleterious effects of some fluid administration.

### Limitations

First, we only observed the short-term effects of PEEP. We judged it was ethically unacceptable to maintain these patients with ARDS at a low PEEP level for a long time. Moreover, it would have been impossible to avoid confounding events (changes in ventilatory setting and fluid administration or removal) over longer periods. Second, we estimated the derecruited volume during the PEEP decrease and not the recruited volume during the PEEP re-increase. Indeed, we speculated that derecruitment may occur faster than recruitment and be easier to detect [34]. Third, we directly measured the PEEP-induced changes in lung volume in 30 patients only, though it is the best method to estimate lung recruitment or derecruitment at the bedside. Estimating derecruitment through changes in Crs, as we did in the whole population, has many limitations [36]. Fourth, the number of saline boluses required for averaging EVLW measurements may have provoked fluid-induced changes in EVLW. However, the fact that EVLW decreased at the first study step indicates that this limitation probably had a very small impact. Fifth, because this was a human study, we could not directly measure the lymphatic flow, a procedure that could have strengthened our conclusions. Finally, we did not insert either a pulmonary artery catheter or an oesophageal balloon and thus could not estimate the hydrostatic lung filtration pressure and the transmural pressure. We thus cannot exclude that changing PEEP also changed the degree of pulmonary oedema formation.

## Conclusions

In ARDS patients, changing the PEEP level induced parallel, small and reversible changes in EVLW. These changes were not due to an artefact of the TPTD technique and are likely due to the PEEP-induced changes in CVP.

## Supplementary information

**Additional file 1: Table S1.** Previous literature regarding positive end-expiratory pressure effects on lung water in acute respiratory distress syndrome.

**Additional file 2: Table S2.** Mean difference of haemodynamic and respiratory variables for post hoc comparisons using Tukey HSD approach.

**Additional file 3: Figure S1.** Possible haemodynamic effects of positive end-expiratory pressure (PEEP) decrease on extravascular lung water (EVLW) levels, not taking into consideration possible artefactual effects related to the transpulmonary thermodilution (TPTD) method.

## Data Availability

Individual, de-identified participant data are available from the corresponding author on reasonable request.

## Abbreviations

ARDS: Acute respiratory distress syndrome
CVP: Central venous pressure
CI: Cardiac index
Crs: Respiratory system compliance
EVLW: Extravascular lung water
FiO_2_: Inspired oxygen fraction
PaO_2_: Arterial oxygen partial pressure
PaO_2_/FiO_2_: Arterial oxygen tension over inspired oxygen fraction ratio
PEEP: Positive end-expiratory pressure
Pplateau: Plateau pressure
PVPI: Pulmonary vascular permeability index
SaO_2_: Arterial oxygen saturation
TPTD: Transpulmonary thermodilution
Vt: Tidal volume
