# Supplementary material for: Transpulmonary thermodilution detects rapid and reversible increases in lung water induced by positive end-expiratory pressure in acute respiratory distress syndrome
Source: Ann Intensive Care. 2020 Mar 2;10:28. doi: 10.1186/s13613-020-0644-2 (PMC7052093; doi:10.1186/s13613-020-0644-2)
Supplement: Supplementary file 1 — Additional file 1: Table S1. Previous literature regarding positive end-expiratory pressure effects on lung water in acute respiratory distress syndrome. [file 13613_2020_644_MOESM1_ESM.doc]

Transpulmonary thermodilution detects rapid and reversible increases in lung water induced by positive end-expiratory pressure in acute respiratory distress syndrome

Francesco GAVELLI, MD; Jean-Louis TEBOUL, MD, PhD; Danila AZZOLINA, PhD; Alexandra BEURTON, MD; Temistocle TACCHERI, MD; Imane ADDA, MD; Christopher LAI, MD; Gian Carlo AVANZI, MD; Xavier MONNET, MD, PhD.

Additional file

**Additional file 1 - Table S1. Previous literature regarding positive end-expiratory pressure effects on lung water in acute respiratory distress syndrome**

| **First author** | **Year of publication** | **Subjects** | **Method of lung water estimation** | **Effect of PEEP increase on lung water** |
| --- | --- | --- | --- | --- |
| Dunegan et al.[9] | 1975 | Animals | DID and G | Decrease |
| Toung et al.[3] | 1977 | Animals | G | Increase |
| Hopewell et al.[18] | 1979 | Animals | NA | No change |
| Miller et al.[19] | 1981 | Animals | DID and G | No change |
| Peitzman et al.[20] | 1981 | Animals | DID and G | No change |
| Peitzman et al.[21] | 1982 | Animals | DID and G | No change |
| Luce et al.[22] | 1982 | Animals | G and H | No change |
| Russel et al.[10] | 1982 | Animals | G | Decrease |
| Saul et al.[23] | 1982 | Animals | DID and G | No change |
| Helbert et al.[26] | 1983 | Animals | G | No change |
| Luce et al.[24] | 1983 | Animals | G and H | No change |
| Slutsky et al.[25] | 1983 | Animals | DID | No change |
| Malo et al.[27] | 1984 | Animals | G and H | No change |
| Carlile et al.[4] | 1986 | Animals | DID and G | Increase |
| Myers et al.[11] | 1987 | Animals | DID | Decrease |
| Borg et al.[12] | 1987 | Animals | DID | Decrease |
| Myers et al.[13] | 1988 | Animals | DID | Decrease |
| Nieman et al.[5] | 1990 | Animals | G | Increase |
| Corbridge et al.[14] | 1990 | Animals | G | Decrease |
| Colmenero-Ruiz et al.[15] | 1997 | Animals | DID and G | Decrease |
| Ruiz-Bailén et al.[16] | 1999 | Animals | G | Decrease |
| Luecke et al.[17] | 2003 | Animals | DID and CT | Decrease |
| Szakmany et al.[6] | 2004 | Humans | TPTD | Increase |
| Toth et al.[28] | 2007 | Humans | TPTD | No change |
| Krebs et al.[7] | 2009 | Humans | TPTD | Increase |
| Wu et al.[8] | 2019 | Animals | TPTD | Increase |

CT: Computed tomography scan, DID: double-indicator dilution, G: gravimetry, H: histology, NA: not available, TPTD: transpulmonary thermodilution
