# Supplementary material for: Transpulmonary thermodilution detects rapid and reversible increases in lung water induced by positive end-expiratory pressure in acute respiratory distress syndrome
Source: Ann Intensive Care. 2020 Mar 2;10:28. doi: 10.1186/s13613-020-0644-2 (PMC7052093; doi:10.1186/s13613-020-0644-2)
Supplement: Supplementary file 2 — Additional file 2: Table S2. Mean difference of haemodynamic and respiratory variables for post hoc comparisons using Tukey HSD approach. [file 13613_2020_644_MOESM2_ESM.doc]

**Additional file 2 – Table S2 - Mean difference of haemodynamic and respiratory variables for post-hoc comparisons using Tukey HSD approach.**

|  | **Comparisons** | | | | | |
| --- | --- | --- | --- | --- | --- | --- |
|  | **High-PEEP_start_ *vs:*** | | | **Low-PEEP_45_ *vs:*** | | |
| **Variables** | **Low-PEEP_15’_** | **Low-PEEP_45’_** | **High-PEEP_end_** | **High-PEEP_start_** | **Low-PEEP_15’_** | **High-PEEP_end_** |
| **Heart rate (min^-1^)** | 1 | 2 | 2 | -2 | -1 | 0 |
| **Systolic arterial pressure (mmHg)** | 2 | -1 | -6 | 1 | 3 | -5 |
| **Diastolic arterial pressure (mmHg)** | -2 | -3 | -3 | 3 | 1 | 0 |
| **Mean arterial pressure (mmHg)** | 0 | -1 | -3 | 1 | 1 | -2 |
| **Central venous pressure (mmHg)** | -3* | -3* | 0 | 3* | 0 | 3* |
| **Cardiac index (L/min/m^2^)** | 0.31* | 0.35* | 0.04 | -0.35* | -0.04 | -0.31* |
| **Cardiac function index (min^-1^)** | 0.3* | 0.3* | 0.1 | -0.3* | 0 | -0.2* |
| **Global end-diastolic volume indexed (mL/m^2^)** | 37* | 41* | -2 | -41* | -4 | -43* |
| **Extravascular lung water (mL/kg)** | -2* | -2* | 0 | 2* | 0 | 2* |
| **Pulmonary Vascular Permeability Index** | -0.1* | -0.1* | 0 | 0.1* | 0 | 0.1 |
| **PEEP (cmH_2_O)** | -9* | -9* | 0 | 9* | 0 | 9* |
| **Pplateau (cmH_2_O)** | -8* | -8* | 0 | 8* | 0 | 8* |
| **Respiratory system compliance (mL/cmH_2_O)** | -1 | -2 | 0 | 2 | 1 | 2 |
| **SpO_2_ (%)** | -2* | -3* | 0 | 3* | 1 | 3* |
| **SaO_2_ (%)** | -3* | -4* | -1 | 4* | 1 | 3* |
| **PaO_2_/FiO₂ ratio** | -34* | -37* | -6 | 37* | 3 | 31* |

FiO_2_: inspired oxygen fraction, PEEP: positive end-expiratory pressure, Pplateau: plateau pressure, PaO_2_: arterial oxygen partial pressure, SaO_2_: arterial oxygen saturation, SpO_2_: pulse oxygen saturation.

*shows a significant difference at 0.05% level
