# Supplementary material for: Transpulmonary thermodilution detects rapid and reversible increases in lung water induced by positive end-expiratory pressure in acute respiratory distress syndrome
Source: Ann Intensive Care. 2020 Mar 2;10:28. doi: 10.1186/s13613-020-0644-2 (PMC7052093; doi:10.1186/s13613-020-0644-2)
Supplement: Supplementary file 3 — Additional file 3: Figure S1. Possible haemodynamic effects of positive end-expiratory pressure (PEEP) decrease on extravascular lung water (EVLW) levels, not taking into consideration possible artefactual effects related to the transpulmonary thermodilution (TPTD) method. [file 13613_2020_644_MOESM3_ESM.doc]

**Additional file 3 - Figure S1. Possible haemodynamic effects of positive end-expiratory pressure (PEEP) decrease on extravascular lung water (EVLW) levels, not taking into consideration possible artefactual effects related to the transpulmonary thermodilution (TPTD) method.**

CVP: central venous pressure; ITP: intrathoracic pressure; LA: left atrial; RA: right atrial; RV: right ventricular; pulm.: pulmonary; TTP: transpulmonary pressure.
